# Effectiveness of individualized rTMS under sMRI guidance in reducing depressive symptoms and suicidal ideation in adolescents with depressive disorders: an open-label study

**DOI:** 10.3389/fpsyt.2024.1485878

**Published:** 2024-12-20

**Authors:** Yuyong Sun, Xiaoyan Liu, Yi Li, Qianna Zhi, Yong Xia

**Affiliations:** Department of Psychiatry, Affiliated Mental Health Center & Hangzhou Seventh People’s Hospital, Zhejiang University School of Medicine, Hangzhou, Zhejiang, China

**Keywords:** adolescent psychiatry, depressive disorders, suicide, magnetic resonance imaging, transcranial magnetic stimulation

## Abstract

**Background:**

Major Depressive Disorder (MDD) is occurring at a progressively younger age, and suicide is now the second leading cause of death among adolescents with MDD. Studies have shown that structural magnetic resonance imaging (sMRI) can improve the positioning accuracy and anti-depressant effects of repetitive transcranial magnetic stimulation (rTMS), thereby reducing suicidal ideation.

**Objective:**

To compare the efficacy of sMRI-guided rTMS combined with pharmacotherapy, surface 5-cm rTMS positioning combined with pharmacotherapy, and pharmacotherapy alone on reducing depressive symptoms and suicidal ideation (SI) in MDD adolescents.

**Methods:**

This was an open-label study of adjustable-dose pharmacotherapy combined with rTMS for the treatment of depressive symptoms and suicidal ideation in MDD adolescents. The three study groups were as follows: sMRI navigation for individualized rTMS coordinates targeting the dorsolateral prefrontal cortex (DLPFC) and in combination with pharmacotherapy for 10 rTMS sessions over two weeks; surface 5-cm positioning for DLPFC in combination with pharmacotherapy for 10 rTMS sessions over two weeks; pharmacotherapy. All patients received only one type of SSRIs anti-depressant. A total of 123 Chinese adolescents aged 13-18 with MDD were enrolled, and psychological parameters were evaluated in the first and second weeks of treatment.

**Results:**

Following treatment, the clinical symptoms improved in all three groups. The sMRI navigation group exhibited significantly more improvement in depressive symptoms and suicidal ideation, without severe adverse reactions.

**Conclusion:**

Ten sessions of rTMS treatment are feasible and effective in improving depressive symptoms and reducing SI in MDD adolescents. The combination of sMRI navigation rTMS and pharmacotherapy was found to yield the best outcomes.

**Clinical trial registration:**

https://www.medicalresearch.org.cn/index, identifier MR-33-24-030536.

## 1 Introduction

Major depressive disorder (MDD) is a severe condition acknowledged worldwide as a major factor for disability and functional impairment (1). Adolescence and young adulthood are peak periods for the onset of major depressive disorder (MDD) (2). Early-onset depression negatively impacts physical and mental development and is associated with poorer academic, occupational, and social achievements (3). Furthermore, depressive episodes during adolescence are linked to an increased risk of suicide, psychiatric and medical comorbidities, as well as a heightened risk of major depressive and anxiety episodes later in life (4). Suicide has been identified as the second most prevalent cause of death among adolescents with MDD (5). To mitigate the impact of depression among young individuals, it is essential to select treatments that are both effective and safe, while also identifying vulnerability factors early in MDD to inform targeted prevention and early intervention strategies (6, 7). Despite the proven effectiveness of pharmacotherapy and psychotherapy (8), challenges remain with the use of pharmacotherapy in clinical practice (9). These include poor acceptance by parents, low medication adherence, limited drug choices for adolescents, and the potential risk of suicide associated with the use of anti-depressants (10). Although the therapeutic benefits of modified electroconvulsive therapy (MECT) have been reported, there is still a lack of comprehensive research on its application in adolescent patients (11). The efficacy, risk-benefit ratio, and ethical considerations regarding informed consent for MECT in this population are the subject of ongoing controversy (12). Consequently, there is a pressing need to explore alternative treatment approaches that are both safe and effective in improving depressive symptoms in minors with MDD.

Repetitive Transcranial Magnetic Stimulation (rTMS) is a non-invasive therapeutic approach. rTMS is based on the physical principle of electromagnetic induction, whereby a magnetic field is generated through a transcranial magnetic stimulation coil. This leads to the production of an induced electric field within the cerebral cortex that is sufficient to elicit action potentials in neurons within the targeted brain region. In 2008, the Food and Drug Administration (FDA) approved application of 10 Hz high-frequency rTMS to the left dorsolateral prefrontal cortex (DLPFC) in adults with treatment-resistant depression (13). The DLPFC is primarily associated with negative thought patterns and feelings of hopelessness. Dysfunction in this region is an integral part of the pathophysiology of MDD and may lead to deficits in negative cognitive regulation (14). The activity of the DLPFC is often suppressed in patients with depression. Numerous clinical studies have demonstrated that rTMS at this site can help to restore the function of DLPFC, thereby improving mood and depressive symptoms (15). In addition to improving depressive symptoms, some studies have also shown that rTMS on the DLPFC can significantly decrease suicidal ideation in MDD patients (16). Several systematic reviews have concluded that traditional rTMS based on the 5-cm positioning method is a valuable therapeutic option for adolescent depression (17, 18). A series of high-frequency rTMS is effective for the treatment of depressive disorders in adolescents, while also significantly reducing SI. Zhang (19) reported this treatment has superior anti-depressant efficacy in adolescents compared to adults. In the evaluation of stimulation parameters, the study by Wall indicated that applying 10Hz rTMS at 120% of the motor threshold to the left DLPFC can effectively reduce the severity of depression in adolescents, without serious adverse effects (20). High-frequency rTMS can alter the inhibition-excitation imbalance associated with MDD in the cortical and limbic brain areas. This results in better efficacy against depression and reduced SI, with good tolerance and no significant adverse reactions (21).

The clinical efficacy of rTMS is known to be influenced by stimulation parameters such as the target site and the intensity, duration, and frequency of stimulation (22). The most commonly used strategy to position the DLPFC target site is to place the TMS coil 5 cm anterior to the primary motor cortex representing the hand, with measurement along the scalp curvature. This is known as the 5-cm rule. However, this method may be inaccurate for several reasons (23). The use of an absolute distance such as 5 cm introduces significant bias related to head size, since larger heads have a greater distance between the motor cortex and the DLPFC. Herwig et al. showed that the “5-cm rule” accurately positions the DLPFC target site in only about 30% of cases, and often positions the site behind the DLPFC (24). Structural Magnetic Resonance Imaging (sMRI)-based rTMS effectively addresses the inaccuracy of the surface 5-cm positioning method and has been adopted for head positioning treatment in adult MDD patients. A systematic review found that sMRI-guided TMS treatment gave a response rate of 15% to 83% in MDD adults, while also reducing SI (25–27). However, studies on MRI-guided rTMS treatment are very limited for adolescents. Suicidal behavior in adolescent patients underscores the need to explore more optimized treatment plans in clinical settings (28). With this background in mind and based on high-frequency rTMS positioning in MDD adults, we evaluated the efficacy of sMRI-guided positioning combined with pharmacotherapy for the treatment of depressive symptoms and SI in MDD adolescents. This approach was compared to surface 5-cm-guided rTMS positioning combined with pharmacotherapy, and pharmacotherapy alone. The results of this study provide new insights for the individualized treatment of MDD adolescents.

## 2 Method

### 2.1 Inclusion criteria

From April 2022 to December 2023, MDD patients were recruited at the Hangzhou Seventh People’s Hospital according to the following inclusion criteria: (1) age 13-18 years (inclusive); (2) meet the criteria for current MDD according to the Diagnostic and Statistical Manual of Mental Disorders, Fourth Edition (DSM-IV), as assessed by two professional psychiatrists using the Mini International Neuropsychiatric Interview (MINI); (3) Han ethnicity; (4) right-handed; (5) score of ≥14 on the 17-item Hamilton Depression Rating Scale (HAMD-17); (6) score of non-zero for items four and five of the Beck Scale for Suicide Ideation-Chinese Version (BSI-CV); (7) no medication taken for two weeks prior to enrollment, and only one type of SSRIs anti-depressant used after enrollment, with no change in the type of medication, although dosage adjustments were made based on clinical conditions. The exclusion criteria were as follows: (1) contraindications for MRI, such as metal implants; (2) complication with severe somatic disease, including inflammatory diseases and autoimmune diseases; (3) current or past neurological diseases or history of brain trauma; (4) history of manic or hypomanic episodes. Upon enrollment, all patients must undergo the Structured Clinical Interview for DSM (SCID) assessment. This study was approved by the Ethics Committee of the Hangzhou Seventh People’s Hospital [ethics approval number (078) of 2022]. The study followed the guidelines of the Declaration of Helsinki. Prior to the study, all participants and their parents or legal guardians provided written informed consent.

### 2.2 Enrollment process

All enrolled MDD adolescents were allocated of their own free will into one of three groups with open labels: sMRI-guided positioning combined with pharmacotherapy (sMRI navigation group), surface 5-cm positioning combined with pharmacotherapy (5-cm positioning group), and pharmacotherapy alone(pharmacotherapy group). All participants in the sMRI navigation and 5-cm positioning groups received rTMS treatment once per day and five times per week (total of 10 rTMS sessions over 2 weeks) in conjunction with pharmacotherapy, while the pharmacotherapy group received only the pharmacotherapy regimen. The type of anti-depressant medication taken during the treatment period was not altered, with the medication dosage adjusted according to the clinical condition. In addition, patients with sleep disorders were also given benzodiazepine-class sleep aids.

### 2.3 sMRI precise positioning

**Figure 1:**
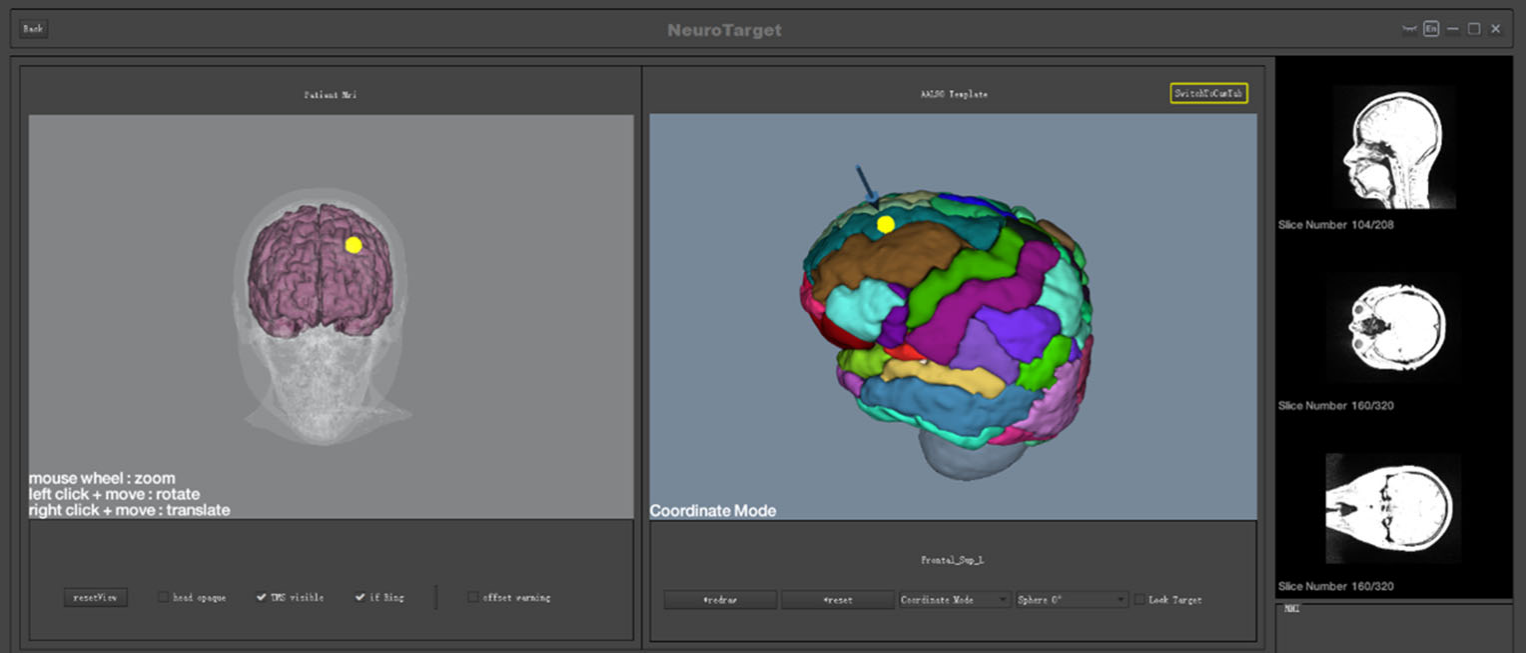
sMRI precise positioning.

Patients received an MRI scan at baseline. MRI data were acquired using the MAGNETOM Prisma 3.0T MRI system and included three-dimensional (3D), high-resolution T1-weighted images and dynamic imaging of brain function of the scalp and 1-mm brain slices. Individual MRI structural and functional images were used to build the 3D model and to calculate the TMS target area. 3D spatial coordinates of individual target areas were derived using brain region templates. The relative position of TMS on the brain was determined by deep learning and image registration with the help of a navigational 3D camera and TMS locator. The TMS target site and TMS coil can be monitored visually after 3D rendering, and the coil focus can be oriented in real-time to the target site. This enables precise navigational positioning and TMS treatment (**Figure 1**).

### 2.4 rTMS protocol

This study utilized a RT-100 magnetic field stimulator (Sichuan Junjian Wanfeng Medical Equipment) equipped with an integrated liquid cooling system (internal circulation) and a figure-eight coil (model CB085A, coil specification 85 mm). The resting motor threshold (RMT) for each participant was determined according to standard clinical practice. The stimulation site was the DLPFC, with the sMRI navigation group targeting the MNI coordinates (-26; 44; 49), and the 5-cm positioning group targeting 5 cm anterior to the left M1. RTMS was conducted at 90% RMT intensity and 10Hz for a total of 2400 pulses. This included a sequence of 4 seconds on and 16 seconds off, for 19 minutes and 44 seconds. The treatment comprised 10 sessions, with one session per day. RMT was defined as the minimum rTMS intensity required to induce a motor response in the contralateral resting abductor pollicis brevis muscle.

### 2.5 Clinical assessment

The HAMD-17 scale and subscale score was used as the primary outcome measure and was assessed at baseline, after 5 sessions of rTMS (1 week), and after 10 sessions of rTMS (2 weeks). The BSI-CV and subscale was used as the secondary outcome measure and includes the intensity of SI and the risk of suicide. BSI-CV was also assessed at baseline and after 1 week and 2 weeks of rTMS. The results for the timeline “at the most despondent, most depressed” in the BSI-CV scale were almost identical to those prior to treatment and thus did not show a treatment effect. Data from the second assessment were therefore analyzed on the “past week” timeline. Taking into account the impact of medication before and after treatment, we used changes in medication dosage as a covariate. Based on the literature (29), we equated 100 mg of sertraline to 20 mg of fluoxetine and 10 mg of escitalopram. This study also compared the treatment efficacy among three groups of MDD adolescents, where treatment responders or those with effective treatment were defined as individuals with a reduction of 50% or more in HAMD-17 scores post-treatment.

### 2.6 Statistical analysis

Statistical analyses were conducted using SPSS 27.0 (IBM, USA) and GraphPad Prism 7.0 (GraphPad Software). Categorical variables were presented as frequency and proportion, with comparisons between groups were made using the chi-square test. Continuous variables with a normal distribution were reported as means and standard deviations. A two-way repeated measures ANOVA was used to analyze main and interaction effects across three treatment groups and three time points for all outcomes. Additionally, the difference scores from baseline were assessed using the same method. *Post hoc* pairwise comparisons were performed with Bonferroni adjustments for multiple comparisons, applying a significance level of α=0.05. The Bonferroni-adjusted significance level for each comparison was set at 0.016.

## 3 Results

### 3.1 Clinical and behavioral characteristics

All patients met the preliminary diagnosis of MDD. A total of 130 patients were screened, of which four patients failed the screening, two failed to complete the MRI examination, and one withdrew from the study due to early discharge after screening. Of the final 123 enrolled patients, 44 were complicated with anxiety disorders, one with obsessive-compulsive disorder, and one with attention deficit disorder. The final analysis included 33 patients in the sMRI navigation group, 41 in the 5-cm positioning group, and 49 in the pharmacotherapy group. With all three groups, assessments were carried out at baseline and after the first and second weeks of enrollment. The three groups showed no significant differences (P > 0.05) in baseline clinical characteristics such as age, gender, education level, disease duration, and family history of mental illness (**Table 1**). All enrolled patients took only one kind of anti-depressant medication during the rTMS period, with 113 taking Selective Serotonin Reuptake Inhibitors (SSRIs), 74 taking sertraline (dosage range 50-150 mg), 31 taking fluoxetine (dosage range 20-60 mg), 18 taking escitalopram (dosage range 10-20 mg), and 75 patients taking benzodiazepine sleep medication in addition.

**Table 1:** Clinical characteristics of the sMRI navigation, 5-cm positioning, and pharmacotherapy groups.

	sMRI navigation	5-cm positioning	Pharmacotherapy	P
	n=33	n=41	n=49	
Age (years)	16.52 ± 2.76	16.41 ± 2.53	16.14 ± 1.35	0.068^a^
Gender (male/female)%	8(24.2)/25(75.8)	9(21.9)/32(78.1)	11(22.0)/38(78.0)	0.971^b^
Education (years)	7.97 ± 1.81	7.46 ± 1.61	7.78 ± 1.55	0.408^a^
Disease duration (months)	10.78 ± 4.39	11.65 ± 3.58	10.89 ± 3.79	0.556^a^
Family history (positive/negative)%	3(9.0)/30(91.0)	6(14.6)/35(85.4)	7(14.2)/42(85.8)	0.736^b^

a One-way ANOVA.

b Chi-square test.

### 3.2 Main effect test of HAMD total score and its factor score

**Figure 2:**
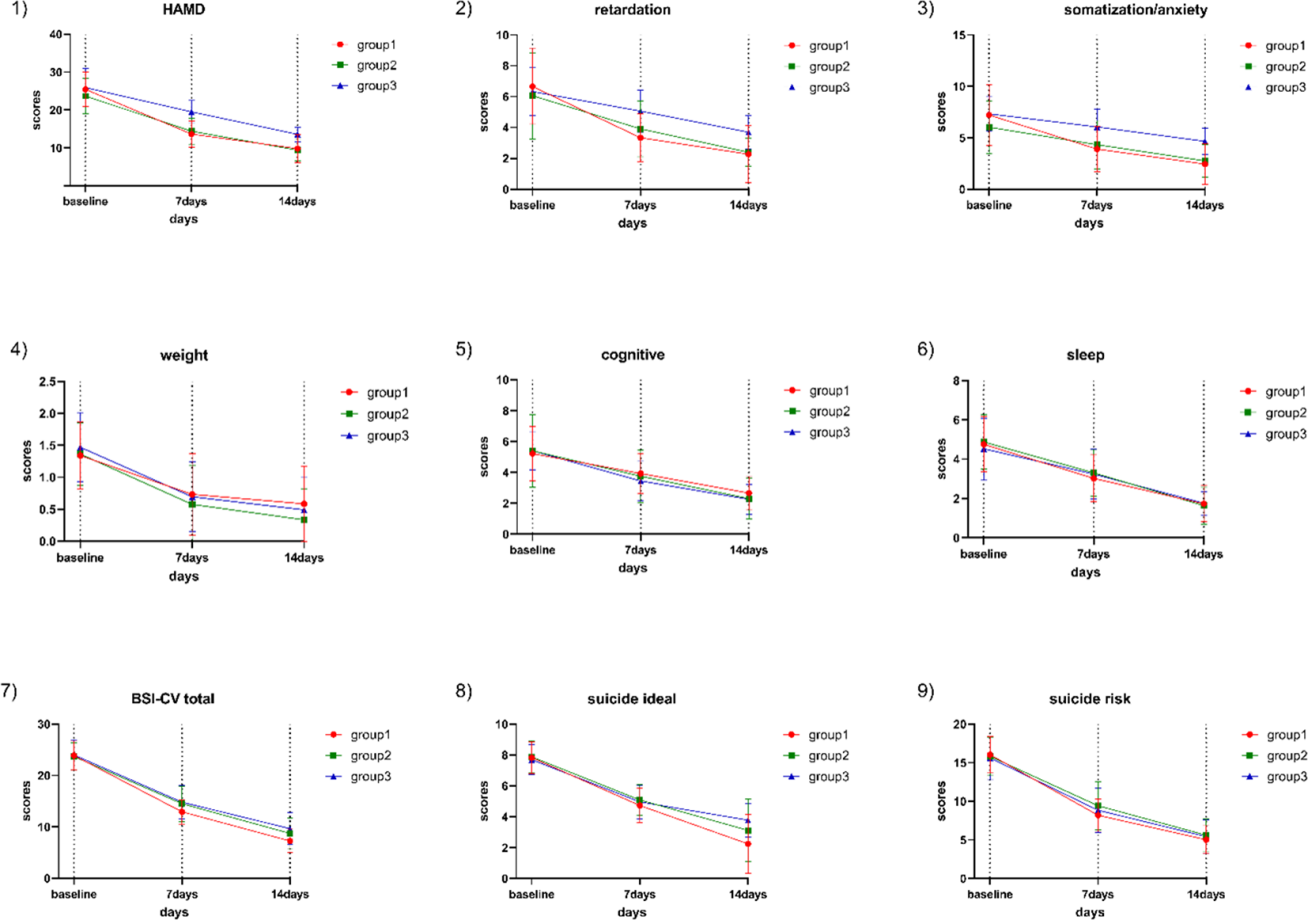
HAMD total score, subscale scores, BSI-CV total score, SI intensity, and suicide risk score in the three groups at baseline and after one and two weeks of treatment. Group 1: sMRI navigation group; Group 2: 5-cm positioning group; Group 3: pharmacotherapy group; 1) Trend in HAMD-17 score changes; 2) Trend in HAMD-17 retardation factor score changes; 3) Trend in HAMD-17 anxiety/somatization factor score changes; 4) Trend in HAMD-17 weight change factor score changes; 5) Trend in HAMD-24 cognitive impairment factor score changes; 6) Trend in HAMD-17 sleep disturbance factor score changes; 7) Trend in BSI-CV score changes; 8) Trend in SI score changes; 9) Trend in suicide risk score changes.

Normal and variance homogeneity tests were performed on the total score and factor scores of HAMD in the 3 groups at different time points during treatment. The data that met the normal test were tested by two-way repeated measures ANOVA. After 2 weeks of treatment, the HAMD scores and their subscale scores were all decreased in the sMRI navigation group, the 5-cm positioning, and the pharmacotherapy group compared to baseline. All emotional scores and their subscale scores were significantly reduced as the corresponding treatments progressed (**Figure 2**; **Table 2**). There was significant interaction between time and grouping in HAMD score (F=11.223, p<0.001), somatic/anxiety factor (F=4.151,p<0.001) and retarding factor score (F=7.262, p<0.001).These results indicate that the time factor has different effect on the emotional state of patients with different treatment methods. Therefore, the time effect and the intergroup effect were tested separately for the three groups of patients.

**Table 2:** Comparison of scale scores between the three groups at baseline and after treatment.

Scale	Group	Baseline	First week	Second week	F	P
HAMD-17 total score	SMRI navigation	25.24 ± 4.944	12.58 ± 2.750^①③⑤⑥^	8.48 ± 2.852^①②⑤⑥^	96.828	**<0.001**
	5-cm positioning	24.17 ± 4.410	15.15 ± 3.511^①③④⑥^	10.59 ± 3.493^①②④⑥^	22.013	**<0.001**
	Pharmacotherapy	25.92 ± 5.024	19.51 ± 3.015^①③④⑤^	13.59 ± 1.936^①②④⑤^	0.343	**<0.001**
	F	1.483	35.699	23.841		
	P	0.231	**<0.001**	**<0.001**		
Somatization/Anxiety subscale score	SMRI navigation	6.88 ± 2.859	3.242 ± 0.308^①③⑤⑥^	1.697 ± 0.220^①②⑤⑥^	13.089	**<0.001**
	5-cm positioning	6.54 ± 2.829	4.818 ± 0.427^①③④⑥^	3.394 ± 0.334^①②④⑥^	0.557	**<0.001**
	Pharmacotherapy	7.33 ± 1.651	6.48 ± 0.262^①③④⑤^	6.48 ± 0.262 ^①②④⑤^	0.24	**<0.001**
	F	1.189	25.330	37.195		
	p	0.308^c^	**<0.001**	**<0.001**		
Weight change score	SMRI navigation	1.36 ± 0.489	0.64 ± 0.103	0.51 ± 0.149	9.191	**<0.001^a^**
	5-cm positioning	1.34 ± 0.530	0.68 ± 0.150	0.68 ± 0.150	7.509	**<0.001**
	Pharmacotherapy	1.47 ± 0.544	0.69 ± 0.121	0.49 ± 0.114	11.636	**<0.001**
	F	0.762	1.584	6.191		
	P	0.469	0.209	0.398		
Cognitive impairment score	SMRI navigation	5.06 ± 1.676	3.64 ± 1.055	2.33 ± 0.777	0.173	**<0.001**
	5-cm positioning	5.49 ± 2.293	4.02 ± 1.753	2.63 ± 0.428	0.046	**<0.001**
	Pharmacotherapy	5.41 ± 1.257	3.45 ± 1.276	2.27 ± 0.974	11.544	**<0.001**
	F	0.590	6.594	4.301		
	P	0.556	0.153	0.265		
Retardation score	SMRI navigation	6.76 ± 2.634	3.273 ± 0.280^①③⑥^	2.152 ± 0.335^①②⑥^	25.691	**<0.001**
	5-cm positioning	6.10 ± 2.577	3.576 ± 0.254^⑥^	2.576 ± 0.185^⑥^	3.715	**<0.001**
	Pharmacotherapy	6.576 ± 0.268	5.455 ± 0.243^①③④⑤^	3.939 ± 0.194^①②④⑤^	0.32	**<0.001**
	F	1.676	18.531	13.371		
	P	0.191	**<0.001**	**<0.001**		
Sleep disorder score	SMRI navigation	4.88 ± 1.341	3.09 ± 0.980	1.67 ± 0.924	1.71	**<0.001**
	5-cm positioning	4.76 ± 1.445	3.20 ± 1.346	1.71 ± 0.655	0.032	**<0.001**
	Pharmacotherapy	4.53 ± 1.569	3.24 ± 1.283	1.76 ± 0.596	1.581	**<0.001**
	F	0.599	0.311	0.373		
	P	0.551	0.733	0.689		

Two-way repeated measures ANOVA.

① Compared with baseline score in the group, P<0.16.

② Compared with 1 week after treatment, P<0.16.

③ Compared with 2 weeks after treatment, P <0.16.

④ Compared with the sMRI navigation group, p<0.16.

⑤ Compared with the 5-cm positioning group, p<0.16.

⑥ Compared with the Pharmacotherapy group, p<0.16.

Bolded P-values indicate statistical significance (P < 0.016).

### 3.3 Individual effect test of HAMD total score and subscale scores

Two-way repeated measures ANOVA was used to compare the changes in HAMD scores and subscale scores at baseline, 1 week and 2 weeks within the three groups. There were no statistically significant differences in the total HAMD scores and its subscale scores among the three groups of patients at baseline (P>0.05). The total HAMD score and its subscale scores were compared within each of the three groups, showing significant differences: baseline > 1 week after treatment >2 weeks after treatment (P<0.001). At the end of the first week of treatment, the total HAMD scores in the sMRI navigation group were lower than those in the 5-cm positioning group (P <0.001) and pharmacotherapy alone group (P <0.001), and the scores in the 5-cm positioning group were lower than those in the pharmacotherapy alone group (P <0.001). At the end of the first week, the sMRI navigation group had lower anxiety/somatization scores than both the 5-cm positioning group and the pharmacotherapy alone group (P<0.001 for both), and the 5-cm positioning group scored lower than the pharmacotherapy alone group (P <0.001). For the retardation score, both the sMRI navigation group and the 5-cm positioning group scored lower than the pharmacotherapy alone group (P <0.001), but there was no significant difference between the sMRI navigation group and the 5-cm positioning group (P =0.092). At the end of the second week, the sMRI navigation group had lower total HAMD and anxiety/somatization scores than both the 5-cm positioning and pharmacotherapy alone groups (P <0.001 for all comparisons). The 5-cm positioning group also scored lower than the pharmacotherapy alone group (P <0.001). For retardation scores, both the sMRI navigation and 5-cm positioning groups scored lower than the pharmacotherapy alone group (P <0.001), but there was no significant difference between the sMRI navigation and 5-cm positioning groups (P =0.086). More details can be found in **Table 2** and **Figure 2**.

### 3.4 Comparison of antidepressant treatment efficacy in MDD patients

**Figure 3:**
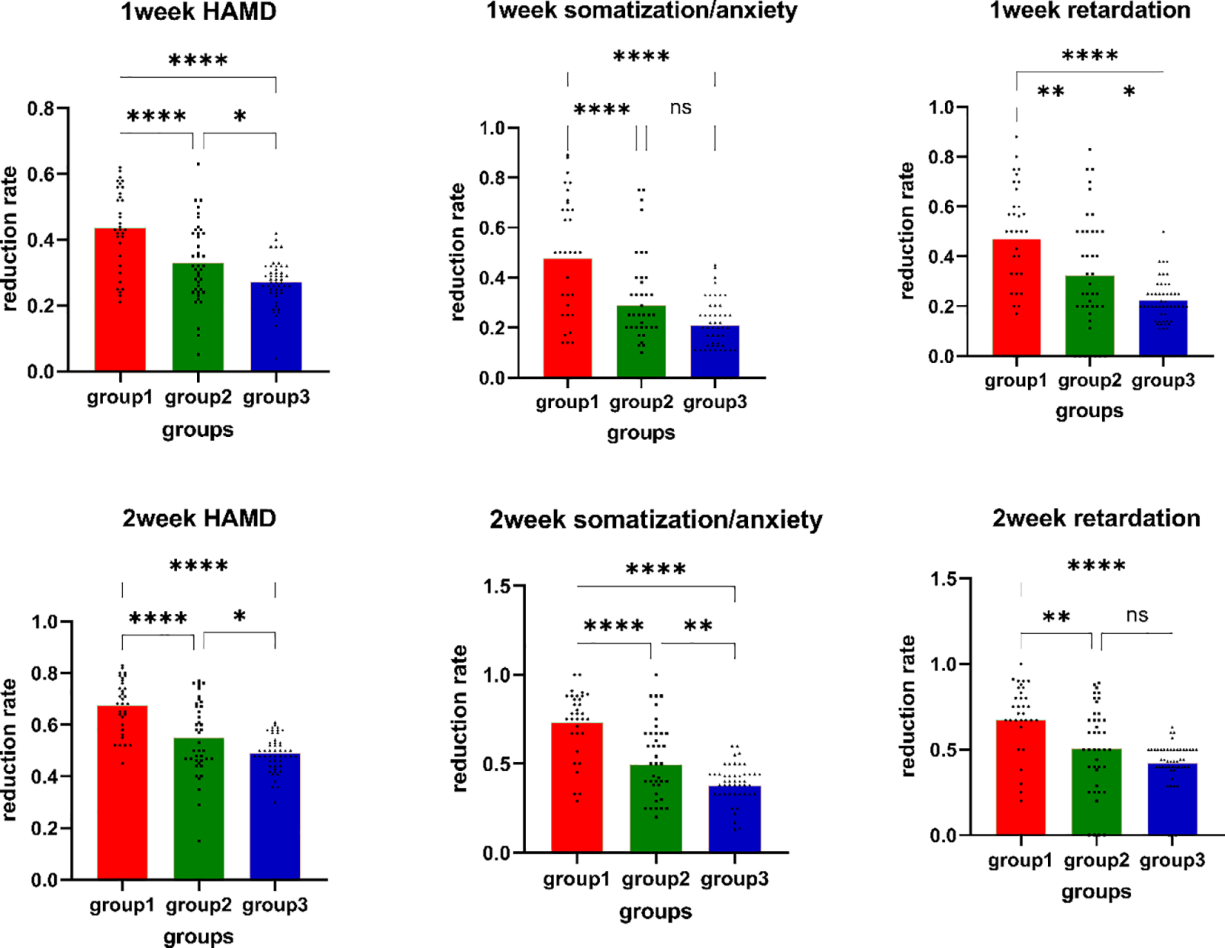
Reduction rates of HAMD-17 total scores, retardation scores, and anxiety scores. Group1: sMRI navigation group; Group2: 5-cm positioning group; Group3: pharmacotherapy group; *p<0.05; **p<0.01; *** p<0.001; **** p<0.0001; NS P>0.05; 1) Week 1 HAMD-17 total score reduction rate comparison; 2) Week 1 somatization/anxiety reduction rate comparison; 3) Week 1 retardation reduction rate comparison; 4) Week 2 HAMD-17 total score reduction rate comparison; 5) Week 2 somatization/anxiety reduction rate comparison; 6) Week 2 retardation reduction rate comparison.

The proportion of treatment responders in the sMRI navigation group reached (57.57%, 19/33) in the first week, which was higher than that in the 5-cm positioning group (31.70%, 13/41) and the pharmacotherapy group (20.0%, 10/49), showing a significant difference in treatment efficacy among the three groups (p=0.002). In the second week of treatment, the proportion of treatment responders in the sMRI navigation group increased to 84.84% (28/33), which was higher than that in the 5-cm positioning group (60.97%, 25/41) and the pharmacotherapy group (46%, 23/49), with significant differences in treatment efficacy among the groups (p=0.002). Further comparison revealed significant differences in the reduction rates of the retardation subscale scores (p<0.001) and the somatization/anxiety subscale scores (p<0.001) among the groups in the first week. In the second week, significant differences were found among the groups [retardation subscale (p<0.001); somatization/anxiety subscale: (p<0.001)]. *Post-hoc* pairwise tests revealed that the differences in reduction rates primarily stemmed from comparisons between the sMRI navigation group with the 5-cm positioning and pharmacotherapy groups. The sMRI navigation group showed significantly higher reduction rates in the somatization/anxiety and retardation subscale scores in both the first and second weeks. More details can be found in **Figure 3**.

### 3.5 Main effect test of BSI-CV total score and subscale scores

Normality and variance homogeneity test were performed on the total BSI-CV score and subscale scores of the 3 groups of patients at different time points during treatment, and the results showed that the total BSI-CV score, SI intensity and suicide risk score of the 3 groups of patients all followed normal distribution and variance homogeneity (P >0.05). The baseline BSI-CV scores among the three groups were not significantly different, indicating no statistically significant differences in suicide at baseline among the three groups (p>0.05). The results showed that time and group had significant interaction on BSI-CV total score (F= 2.031, P <0.001)and SI intensity(F= 7.626, P <0.001). It shows that the effect of time on SI intensity with different treatment methods. Therefore, the time effect and the intergroup effect were tested separately for the three groups of patients.

### 3.6 Individual effect test of BSI-CV total score and its subscale scores

After 2 weeks of treatment, the BSI-CV score and their subscale scores were all decreased in the sMRI navigation group, the 5-cm group, and the pharmacotherapy group compared to baseline. All subscale scores were significantly reduced as the corresponding treatments progressed (**Figure 2**; **Table 2**). There were no significant differences in the total BSI score and its factor scores at baseline among the three groups. The results showed that compared with before treatment, the total BSI-CV score and its subscale scores in the 3 groups were significantly reduced with the corresponding treatment progress. The BSI-CV total and subscale scores decreased significantly over time in all groups: baseline>1week (P <0.001) > 2 weeks (P <0.001). Further, the total score of BSI-CV, SI intensity and suicide risk scores of the three groups were compared at the same time point. There were no significant differences between the three groups in BSI-CV total score, SI intensity and suicide risk at first week. At the end of the second week of treatment, the total score of BSI-CV in sMRI navigation was lower pharmacotherapy alone group(P<0.001), there was no significant difference between the other groups(P=0.023). The SI intensity score of sMRI navigation group was significantly lower than 5-cm positioning group (P<0.001) and pharmacotherapy alone group (P<0.001), and there was no significant difference between 5-cm positioning group and pharmacotherapy alone(p=0.672). There was no significant difference in suicide risk among the three groups(P=0.744), detailed results are shown in **Table 3**.

**Table 3:** Comparison of scale scores among the three groups at baseline and after treatment.

Scale	Group	Baseline	First week	Second week	P
BSI-CV total score	SMRI navigation	23.73 ± 2.719	13.09 ± 2.566^①③^	6.94 ± 2.370^①②⑥^	**<0.001**
	5-cm positioning	23.85 ± 2.744	14.07 ± 3.259^①③^	8.70 ± 2.676^①②^	**<0.001**
	Pharmacotherapy	23.98 ± 2.976	14.82 ± 3.225^①③^	9.88 ± 3.276^①②④^	**<0.001**
	F	0.079	1.128	4.523	
	P	0.924	0.327	0.023	
SI intensity	SMRI navigation	7.85 ± 1.121	4.64 ± 0.994^①③^	1.82 ± 0.667^①②⑤⑥^	**<0.001**
	5-cm positioning	7.85 ± 0.963	5.10 ± 1.114^①③^	3.29 ± 1.028^①②④^	**<0.001**
	Pharmacotherapy	7.69 ± 0.962	4.96 ± 1.098^①③^	3.78 ± 1.104^①②④^	**<0.001**
	F	0.360	1.737	11.834	
	P	0.698	0.180	**<0.001**	
Suicide risk	SMRI navigation	15.88 ± 2.329	8.45 ± 2.209^①③^	5.12 ± 1.900^①②^	**<0.001**
	5-cm positioning	16.00 ± 2.470	8.98 ± 2.954^①③^	5.41 ± 2.000^①②^	**<0.001**
	Pharmacotherapy	15.63 ± 2.804	8.84 ± 2.860^①③^	5.43 ± 2.217^①②^	**<0.001**
	F	0.239	0.628	0.257	
	P	0.788	0.535	0.744	

Two-way repeated Measures ANOVA.

① Compared with baseline score in the group, P<.0.016.

② Compared with 1 week after treatment, P<0.016.

③ Compared with 2 weeks after treatment, P <0.016.

④ Compared with the sMRI navigation group, p<0.016.

⑤ Compared with the 5-cm positioning group, p<0.016.

⑥ Compared with the Pharmacotherapy group, p<0.016.

Bolded P-values indicate statistical significance (P < 0.016).

### 3.7 Assessment of adverse reactions in patients of the sMRI navigation group and the 5-cm positioning group after treatment

When administering rTMS treatment to adolescents with depression, the most common side effects include dizziness and localized headaches, but these symptoms can be alleviated after the treatment concludes. Seizures are the most severe side effect of rTMS, and medication use is a possible cause of seizure induction. Adolescents, as a special group, may have a lower threshold for seizure induction compared to adults, making it crucial to understand the patient’s medication history before conducting rTMS treatment. Additionally, a study pointed out that excessively high stimulation intensity of rTMS can also induce seizures, so timely assessment and adjustment of the stimulation intensity are important measures to ensure the safety of rTMS (30). During the treatment process, among all adolescent patients diagnosed with MDD, three patients reported dizziness after the first rTMS treatment, with an incidence rate of 2.4%. The dizziness they experienced was mild and transient, resolving after a short rest, and did not recur in subsequent treatments. The remaining patients did not report any significant adverse reactions, and no seizures occurred, indicating a general tolerance and acceptance of the treatment.

## 4 Discussion

This study of adolescent MDD patients utilized sMRI-guided rTMS for precise positioning, as well as multidimensional assessment of the emotional state and SI before and after rTMS treatment. A pharmacotherapy group served as the control. The sMRI navigation group showed significant and rapid anti-depressant efficacy, as well as reduced SI. Moreover, adolescent MDD patients showed high tolerance to 10Hz sMRI-navigated rTMS. No severe adverse reactions such as serious headaches, pain at the stimulation site, or epilepsy were encountered during the treatment period, thus demonstrating the safety and tolerability of 10Hz sMRI-guided rTMS treatment in MDD adolescents. In summary, 10Hz sMRI-guided rTMS is a safe, effective, and feasible anti-depressant treatment. This opens a new strategy for the clinical treatment of adolescent MDD.

Analysis of the changes in HAMD-17 scores during treatment revealed a significant interaction between treatment time and group. This suggests the improvement of clinical symptoms in the different treatment groups varied with time. After the first and second weeks of treatment, The sMRI navigation group showed lower scores and greater efficacy than other groups, the rTMS combined with pharmacotherapy groups had significantly lower HAMD scores than the pharmacotherapy alone group.

This suggests that rTMS is effective at stimulating the rapid onset of action of anti-depressant drugs. The standard rTMS protocol approved by the FDA for treating adult depression targets the L-DLPFC with 10Hz stimulation (31). Given that younger age is a favorable factor for the effectiveness of rTMS, researchers have begun to explore the efficacy and safety of rTMS in children and adolescents to develop suitable protocols for these age groups. One study showed that after treatment with 10Hz rTMS, adolescents showed significant improvement in depression symptoms, as indicated by scores on the Children’s Depression Rating Scale-Revised (CDRS-R), and there was also significant improvement in scores on the Clinical Global Impressions-Severity scale (CGI-S) (32). The rTMS treatment conducted by Croarkin on 10 adolescents with treatment-resistant depression showed an increase in the glutamate/glutamine ratio after treatment, which was negatively correlated with the severity of depression. This suggests that rTMS may improve depressive symptoms by modulating glutamate neurotransmission (33). MRI study also found that rTMS can induce changes in the amplitude of low-frequency fluctuations (ALFF), regional homogeneity (ReHo), and functional connectivity strength (FCS) in brain regions associated with depression (34). Research on adults indicates that treatment duration, specifically 10 rTMS sessions within two weeks, was effective at improving depressive symptoms, highlighting the importance of treatment duration for TMS efficacy (35). Consistent with this, the current study also found that minors showed significant improvement in depressive symptoms after 10 rTMS sessions. The combination of rTMS with anti-depressant medication presents notable advantages due to the synergistic effects. Medication improves mood by regulating neurotransmitter levels in the brain, while rTMS exerts therapeutic effects by non-invasively stimulating neural activity in specific brain areas. When used together, rTMS can target brain areas that are otherwise challenging for medication alone, thus providing a more comprehensive treatment approach. The sMRI navigation and 5-cm positioning groups showed higher rates of reduction in the anxiety/somatization scores than the pharmacotherapy group. These scores included depressive mood, loss of interest, anxiety, and somatic anxiety, reflecting the core symptoms of MDD. The combination of rTMS with medication may therefore offer notable benefits in improving depressive symptoms through additive effects, especially by improving retardation and anxiety and enhancing interest in activities.

This study also explored whether rTMS treatment improves SI in MDD adolescents. SI in all three groups improved after treatment, consistent with previous research findings (36–39). Multifactorial repeated measures ANOVA revealed a significant interaction between treatment time and group during the treatment period. This finding suggests differences between the three treatment groups in terms of SI changes. After the second week of treatment, the sMRI navigation group showed significantly lower total BSI-CV scores and SI severity compared to the 5-cm positioning and pharmacotherapy groups. A one-week double-blind study found that neuro navigation-guided high-dose rTMS significantly improved depressive symptoms and reduced suicidal ideation in MDD patients (40).

Furthermore, the SI of patients in the sMRI navigation group decreased notably after the first week of treatment compared to sham stimulation. Additionally, 3 days of high-frequency rTMS on the DLPFC area significantly reduced SI in adult MDD patients (41). The rapid onset of action could be due to the thrice daily 30-minute sessions of high-frequency rTMS, resulting in a total of 54,000 stimuli. rTMS targets the DLPFC to enhance neural activity in this area, thereby improving emotion regulation, cognitive control, and impulse inhibition, which are crucial for reducing suicidal ideation. Currently, adolescents typically do not undergo such high-frequency and high-intensity stimulation protocols due to safety considerations. RTMS can reshape the connectivity of key neural networks in the brain, including the default mode network (DMN) and the central executive network (CEN), optimizing brain functional integration and enhancing emotional and cognitive processing. Neurotransmitter modulation is also an important mechanism of rTMS; by affecting levels of neurotransmitters such as dopamine and glutamate, rTMS provides a biological basis for improving depressive symptoms and reducing suicidal ideation (42). More importantly, rTMS promotes neuroplasticity through repetitive stimulation, leading to adaptive structural and functional changes in the brain. This helps establish healthier emotional and behavioral patterns and enhances patients’ psychological resilience. The combined effects of these mechanisms provide a solid theoretical foundation and empirical support for the use of rTMS in intervening in adolescent suicidal ideation (26).Currently, there is no consensus on the feasibility and safety of multiple daily sessions of rTMS in the adolescent population. Due to safety considerations, we did not apply multiple daily rTMS for our adolescent patients. This exploratory study in adolescents found that SI decreased significantly after 10 sessions of rTMS over 2 weeks. Anti-depressant medications typically take effect after 2 weeks. The current study revealed that rTMS combined with medication can effectively control early-stage SI and improve clinical symptoms after 2 weeks, leading to a substantial reduction in early suicide risk. This suggests that the combined treatment model provides prompt relief of SI in patients, with rapid onset and a high safety profile. SI is one of the strongest predictors of suicidal behavior and usually precedes suicide intention and behavior (43, 44). Active and effective intervention is particularly critical in ensuring the life safety and quality of life of MDD patients. The present study not only provides evidence for the safety of rTMS treatment in adolescents, but also suggests that rTMS is a potential treatment option for reducing SI.

Overall, the sMRI navigation group showed a greater reduction of depressive symptoms and SI than both the surface 5-cm positioning and pharmacotherapy groups. Precision localization of TMS, such as with the use of a neuro-navigation system, is superior to traditional surface localization methods because it allows individualized localization based on sMRI. The combination of sMRI and neuro-navigation for TMS localization enables the reconstruction of head models using participant-specific sMRI data. Compared to the traditional 5-cm positioning method, this results in a better match of the real head shape of the participant (24), thereby reducing heterogeneity due to differences in individual anatomical structures. Precise localization can reduce stimulation of surrounding non-target areas, enhance the efficacy and response rate of rTMS, and thus minimize any side effects. Personalized rTMS/TBS protocols were incorporated with neuroimaging in a retrospective analysis of the therapeutic effect of individualized rTMS for depression (45). By acquiring three-dimensional (3D) T1-weighted structural MRI data from subjects and inputting it into a neuronavigation system, brain structures can be visualized, which has a positive impact on improving the accuracy of target localization. Fitzgerald (46) conducted a randomized controlled trial involving 51 patients with treatment-resistant depression, who were randomly divided into two groups: the “5 cm localization method” group and the neuronavigation group The results showed that the neuronavigation group had better outcomes. This demonstrated the benefits of personalized stimulation parameters for improving clinical outcomes. Such methods require further validation in future clinical trials. During the treatment process, no severe adverse events including severe headaches or epilepsy were observed (47), and no related adverse reactions such as flashes of light, blurred vision, or hallucinations occurred. Indeed, only three patients in the present study experienced dizziness after treatment. This was alleviated after rest, indicating relatively good tolerability and few major side effects from the treatment.

As previously mentioned, the enrolled participants in our study were complicated with other disorders. They were not excluded, however, because their comorbidities reflect the real-world conditions of MDD adolescents (48). Although these comorbid diseases were not assessed here, previous studies have reported on the therapeutic effects of rTMS for obsessive-compulsive disorder and anxiety disorders (49). However, larger study cohorts are required to allow more comprehensive assessments. The risk of adolescents with MDD transitioning to bipolar disorder (BD) is estimated at 45% (50). To address this, we applied strict inclusion criteria, assessing family history and manic symptoms to minimize high-risk cases. We also plan long-term follow-up to monitor BD emergence, identify predictors.

In this study, the sMRI navigation group showed favorable anti-depressant efficacy and reduction of SI compared to the 5-cm positioning and pharmacotherapy groups. This combined approach may be an important way to further enhance the efficacy of treatment for MDD in adolescents. However, this study also had several limitations, including the relatively small sample size. Although information was available for the duration of illness at baseline, there was a lack of data on the number of episodes and the specific medication. Therefore, the current results require confirmation by performing multi-center, randomized controlled trials with a larger sample size. Considering the heterogeneity of MDD, further investigation of different MDD subtypes is also required in order to achieve individualized and precise rTMS intervention strategies. This is necessary for both clinical symptom and biological subtypes, with the goal of providing adequate evidence for the application of rTMS in the treatment of depressive disorders. Additionally, we did not set up sham control treatment after patient enrollment. Anti-suicidal treatment in adolescents remains challenging. In particular, determining the capacity of adolescents to consent to research intervention is a complex process in the context of emotional dysregulation, historically suboptimal decision-making, and recent suicide attempts. Several limitations must be considered in the current study. Moreover, as part of the study design, participants were required to use only one type of anti-depressant medication during treatment and not change the type of medication. Although we inferred the observed clinical changes as being attributable to rTMS rather than to previous treatments, we cannot exclude a possible impact of previous treatments on the clinical outcomes.

## 5 Conclusion

This study employed an open-label design and combined two weeks of high-frequency rTMS treatment with anti-depressant medication. sMRI-guided rTMS treatment was found to be more effective than the traditional 5-cm positioning method and pharmacotherapy. Therefore, 10Hz rTMS based on individualized and precise sMRI of the DLPFC region can improve the clinical symptoms and SI of adolescents with MDD. This combined approach may be a feasible, effective, and safe treatment option for such patients.

## Data Availability

The raw data supporting the conclusions of this article will be made available by the authors, without undue reservation.
